# 

**Published:** 1888-07

**Authors:** 


CONTENTS.

VOL. 35 No. 7.

| | PAGE. |
|---|---|
| The New World Language | 145 |
| Breathing Exercises, Etc. | 146 |
| Mind Cure, No. 3 | 150 |
| Volapük not a correct or Scientific World Language | 155 |
| Printing and Engraving | 158 |
| A Dream Realized | 159 |
| Implanting Teeth | 160 |
| Gettysburg Battle Field | 161 |
| Death of Emperor Frederick III | 163 |
| Book Notices | 164 |
| Household | 165 |
| Miscellaneous | 167 |

INDEX TO ADVERTISEMENTS OF HALF A PAGE AND OVER.

| | PAGE. |
|---|---|
| Crystal Pepsin Tablets | I |
| Yankee Blade | I |
| Stevensville Mills | II |
| The Tea Cure | II |
| The American Oxygen Association | III |
| Hollow Suppositories | IV |
| Lactopeptine | V |
| Premiums of Books | VI |
| Premiums of Silver Plate | VII |
| Bovinine | VIII |
| Volapük | IX |
| Rio Chemical Co. | X |
| The Welsbach Incandescent Gas Burner | XI |

